# Fed-batch fermentation of GM-CSF-producing glycoengineered *Pichia pastoris *under controlled specific growth rate

**DOI:** 10.1186/1475-2859-9-93

**Published:** 2010-11-23

**Authors:** Pieter P Jacobs, Mehmet Inan, Nele Festjens, Jurgen Haustraete, Annelies Van Hecke, Roland Contreras, Michael M Meagher, Nico Callewaert

**Affiliations:** 1 Unit for Molecular Glycobiology, Department for Molecular Biomedical Research, VIB, Ghent, Belgium; 2 Department of Biomedical Molecular Biology, Ghent University, Ghent, Belgium; 3 Current Address: Department of Dermatology, Brigham & Women's Hospital and Harvard Medical School, Boston, MA, USA; 4 Biological Process Development Facility, Department of Chemical and Biomolecular Engineering, University of Nebraska-Lincoln, Lincoln, NE, USA; 5 Protein Service Facility, Department for Molecular Biomedical Research, VIB, Ghent, Belgium; 6 Laboratory for Protein Biochemistry and Biomolecular Engineering, Department of Biochemistry, Physiology and Microbiology, Ghent University, Ghent, Belgium; 7 Current Address: Department of Food Engineering, Akdeniz, University, Antalya, Turkey

## Abstract

**Background:**

Yeast expression systems with altered N-glycosylation are now available to produce glycoproteins with homogenous, defined N-glycans. However, data on the behaviour of these strains in high cell density cultivation are scarce.

**Results:**

Here, we report on cultivations under controlled specific growth rate of a GlycoSwitch-Man5 *Pichia pastoris *strain producing Granulocyte-Macrophage Colony-Stimulating Factor (GM-CSF) at high levels (hundreds of milligrams per liter). We demonstrate that homogenous Man_5_GlcNAc_2 _N-glycosylation of the secreted proteins is achieved at all specific growth rates tested.

**Conclusions:**

Together, these data illustrate that the GlycoSwitch-Man5 *P. pastoris *is a robust production strain for homogenously N-glycosylated proteins.

## Background

Despite the availability of five classes of heterologous protein production platforms (bacteria, yeasts, plants, insect cells, and mammalian cells), more than 50% of currently marketed biopharmaceuticals are produced in mammalian cell lines [1]. This is in part due to the inability of the remaining four classes to modify glycoproteins with human-like oligosaccharides. This is of importance since protein-bound glycans influence circulation half-life, tissue distribution, and biological activity. In addition, they may be immunogenic. Therefore, engineering of the glycosylation pathway of most currently used heterologous protein expression systems is an active field of research [2].

Although many biopharmaceutical companies have been experimenting for many years with the methylotrophic yeast *P. pastoris *as an expression system for the production of therapeutic proteins, the first *Pichia *product approval by the FDA was announced only very recently (KALBITOR, Dyax Corp.). This now established regulatory approval pathway, together with the recent availability of glycoengineered strains that produce heterologous proteins predominantly as single glycoforms [3,4], should pave the way for future biopharmaceutical production. As a result, batch-to-batch variability is reduced and downstream processing is facilitated due to increased product homogeneity. Moreover, pharmacokinetic and pharmacodynamic properties can be more easily controlled and fine-tuned, as has been shown for human IgG [5] and rat erythropoietin [3].

Over the past decade, we have created several glycoengineered *P. pastoris *strains each capable of modifying its glycoproteins with predominantly one defined N-glycan structure [2,4,6,7]. Recently, we have started to explore their behavior under controlled bioreactor conditions. The primary goal of these experiments was to determine the robustness of our strains in terms of N-glycan homogeneity and product yield when subjected to different growth conditions. In this study, we have used a glycoengineered *P. pastoris *strain capable of modifying its glycoproteins with Man_5_GlcNAc_2 _N-glycans to produce murine granulocyte-macrophage colony-stimulating factor (GM-CSF) as a test protein.

GM-CSF belongs to a family of colony-stimulating factors that regulate proliferation and differentiation of hematopoietic cells [8]. In response to inflammatory stimuli, GM-CSF is released by various cell types including T lymphocytes, macrophages, fibroblasts and endothelial cells [9,10]. GM-CSF then activates and enhances the production and survival of neutrophils, eosinophils, and macrophages [11].

In the clinic, GM-CSF is used for treatment of neutropenia and aplastic anemia following chemotherapy and greatly reduces the risk of infection associated with bone marrow transplantation. Its utility in myeloid leukemia treatment and as a vaccine adjuvant is well established [12,13]. The two GM-CSF analogs currently on the market differ from each other and from the native protein on two points, primary structure and glycosylation status. Sargramostim is produced in *S. cerevisiae*. Like native GM-CSF, it has 127 amino acids and is glycosylated but differs from native GM-CSF in molecular mass and in the substitution of leucine for proline at position 23. Therapeutic GM-CSF expressed in *Escherichia coli *is not glycosylated, has six fewer amino acids than the native protein, and an extra methionine at position 1 [14]. *E. coli*-produced GM-CSF is also known as Molgramostim (marketed as Leucomax^® ^by Schering-Plough), but was never approved for use in the United States by the FDA because it was associated with a higher incidence of adverse effects than Sargramostim [14]. The latter product is produced in *S. cerevisiae *and is marketed as Leukine^® ^(Bayer Healthcare Pharmaceuticals).

Murine GM-CSF (mGM-CSF) is a 124 amino acid glycoprotein with an apparent molecular weight of 14-33 kDa [15]. It contains two intramolecular disulfide bonds (Cys51-93, Cys85-118), two potential N-glycosylation sites (Asn66 and 75) as well as sites of O-glycosylation [16]. The protein is very resistant to denaturing and proteolytic conditions [15]. Non-glycosylated GM-CSF is fully biologically active and in fact is up to 10 times more potent than the fully glycosylated protein. The lower *in vitro *biological activity of glycosylated GM-CSF is due to a reduction in receptor binding affininty [17,18].

Here, we describe the design of a basic but robust fed-batch fermentation process for the production of a Man_5_GlcNAc_2 _glycoform of mGM-CSF by a glycoengineered *P. pastoris *strain. This approach allowed for maximal production at high cell densities while maintaining N-glycan homogeneity.

## Materials and methods

### Strains and media

*Escherichia coli *MC1061 was used as the host strain for plasmid propagation. The bacteria were cultivated in LB medium (1% tryptone, 0.5% yeast extract, and 0.5% sodium chloride) containing 100 μg/ml ampicillin.

The *P. pastoris *GlycoSwitch-Man5 strain modifies its glycoproteins predominantly with Man_5_GlcNAc_2 _N-glycans. It only differs from a previously reported GS115-derived strain in the selection marker used to engineer the N-glycosylation pathway, blasticidin instead of zeocine [7].

Small-scale *P. pastoris *cultivation was performed in BMGY (1% yeast extract, 2% peptone, 1.34% yeast nitrogen base, 100 mM potassium phosphate buffer [pH 6.0], 1% glycerol) and BMMY medium (1% yeast extract, 2% peptone, 1.34% yeast nitrogen base, 100 mM potassium phosphate buffer [pH 6.0], 1% methanol).

Fermentations were performed in FM22 medium [19], which is deionized water containing (per liter): 42.9 g KH_2_PO_4_, 5.0 g (NH_4_)_2_SO_4_, 1.0 g CaSO_4_.2H_2_O, 14.3 g K_2_SO_4_, 11.7 g MgSO_4_.7H_2_O, 40.0 g glycerol. Prior to inoculation, the pH was adjusted to 5.0 with concentrated ammonium hydroxide followed by the addition of 8.7 ml of *Pichia *Trace Metals (PTM1; containing, per liter, 6.0 g CuSO_4_._5_H_2_O, 0.08 g NaI, 3.0 g MnSO_4_.H_2_O, 0.2 g Na_2_MoO_4_.2H_2_O, 0.02 g H_3_BO_3_, 0.5 g CoCl_2_, 20.0 g ZnCl_2_, 65 g FeSO_4_.7H_2_O, 0.2 g biotin, and 5.0 ml H_2_SO_4_).

### Vector construction and transformation

The *P. pastoris *expression vector pPIC9mGM-CSF contains the mature mouse GM-CSF coding sequence fused in-frame to the *S. cerevisiae *alfa mating factor signal sequence under transcriptional control of the *AOX1 *promoter. The mGM-CSF ORF was amplified by PCR using pORF9-mGMCSF (Invivogen) as template and the oligonucleotides 5'-CTAGCTCGAGAAAAGAGAGGCTGAAGCCGCACCCACCCGCTCACCC-3' and 5'-AGTTTAGCGGCCGCTCATTTTTGGCCTGGTTTTTTGC-3' as primers (XhoI and NotI restriction sites are underlined). Subsequently, an XhoI/NotI fragment was cloned into the XhoI/NotI opened pPIC9 expression vector (Invitrogen).

Linearized vectors (SalI) were transformed into *P. pastoris *GS115 and GlycoSwitch-Man5 as described [20], resulting in GS115mGM-CSF and Man5mGM-CSF, respectively. Genomic integration was confirmed by performing PCR on genomic DNA.

### Small scale shake flask expression conditions

Cells were grown in 10 ml BMGY medium at 30°C. After 48 hours of cultivation, the cells were pelleted by centrifugation at 3000*g *for 5 minutes and resuspended in BMMY medium. Induction was maintained for another 48 hours by spiking the cultures twice daily with 100 μl of 100% methanol (1% final concentration). Subsequently, the cultures were harvested by centrifugation (3000*g *for 10 minutes) and the supernatants were frozen at -20°C until further use.

### Fermentor setup

Fermentations were carried out in 5-liter BioFlo III or 3000 bioreactors (New Brunswick Scientific) containing two liters of FM22 medium [19] supplemented with 8.7 ml PTM1 trace salts (added after autoclaving). The temperature was maintained at 30°C and the pH at 5.0 (controlled with concentrated ammonium hydroxide). The dissolved oxygen (DO) was set to 40% and controlled by an agitation/O_2 _cascade. Pure oxygen was supplemented as needed to maintain the DO setpoint. A part of the off-gas was diverted to an MC-168 methanol monitor and controller (PTI Instruments) equipped with a TGS822 methanol sensor (Figaro Engineering), which were used to maintain a constant level of methanol in the broth. A methanol feed pump (Model 101U/R, Watson-Marlow), balance (Model PR1203, Mettler Toledo) and the MC-168 controller were interfaced with the NBS-BioCommand supervisory control and data acquisition software to make a closed-loop feed control system. The amount of methanol delivered was measured using a balance as the difference between the initial mass in the methanol tank and the current mass.

The *P. pastoris *strain Man5mGM-CSF was grown in a 1-liter shake flask with 300 ml BMGY medium for 20-24 h at 30°C and 300 rpm to an optical density (at 600 nm) of 4-8. Following microscopic examination for contamination, 100 ml of this pre-culture was used as inoculum.

To increase cell mass and simultaneously prepare the cells for induction, a glycerol fed-batch phase was performed. A 1-hour fed-batch period was used at a feed rate of 20 g 50% w/w glycerol (containing 12 ml PTM1 trace salts per liter) per hour per liter of broth. Following the fed-batch phase, a transition phase was applied to shorten the time required for the cells to fully adapt to methanol. The transition phase was initiated by the addition of 1.5 g/l methanol. Simultaneously, the glycerol feed rate was ramped down linearly from 20 g/l/h to 0 over a 3-h period. By the end of the transition phase, the cells were fully adapted to methanol, which was confirmed by a sharp drop in DO [21].

Once the cells were fully adapted to methanol, the methanol fed-batch phase was started. Methanol supplemented with 12 ml of PTM1 per liter was used. Two feeding strategies were applied. The first was a methanol-excess feed strategy in which the methanol concentration was kept constant at ~2 g/l. This allowed the maximum specific growth rate (μ_max_) to be determined. The second strategy used methanol-limited feeding to maintain the growth rate below μ_max _at a constant predetermined value. This was achieved by varying the methanol feed rate (F) exponentially, using the following equation [21]:

(1) $$\text{F}=\nu_{\text{MeOH}}({\text{X}}_{0}{\text{V}}_{0}){\text{e}}^{\mu \text{t}}$$

X_0 _is the cell density and V_0 _the broth volume at the start of the feeding profile (t = 0), μ is the desired specific growth rate (h^-1^), and ν_MeOH _is the specific methanol consumption rate (g/g/h). The latter can be calculated once μ_max _and ν_MeOH, max _have been determined empirically under methanol excess conditions. For a desired μ, ν_MeOH _is estimated by the following equation:

(2) $$\nu_{\text{MeOH}}=\mu \nu_{\text{MeOH},\max}/\mu_{\max}$$

Equation (2) is based on the assumption that the yield of biomass to substrate Y_X/S _= μ_max_/ν_MeOH,max _and is independent of μ and that the maintenance coefficient is negligible [21].

### Protein purification

All purification steps were conducted at 4°C. A GS115 mGM-CSF-secreting clone was grown in BMGY-containing shake flasks at 30°C. After 24 hours of cultivation, the cells were harvested by centrifugation and mGM-CSF protein expression was induced by resuspending the cell pellet in BMMY medium containing 2% methanol. At 24, 36 and 48 hours of induction, 2% methanol was added to maintain induction. After 60 hours, five liter of cultivation medium was then obtained upon centrifugation at 18,000*g *for 30 minutes. Ammonium sulphate was added to 80% saturation and the suspension was incubated overnight to precipitate the protein fraction. The protein pellet was isolated by centrifugation at 18,000*g *for 30 minutes and redissolved in 90 ml of 25 mM sodium acetate pH 4.5, 0.1% CHAPS. After centrifugation at 40,000*g *for 30 minutes to remove a minor non-soluble fraction, this solution was desalted on a Sephadex G25 column of 475 ml (XK26 × 90 cm, GE Healthcare) to 25 mM sodium acetate pH 4.5, 0.1% CHAPS. The desalted protein fraction was loaded on a 20 ml Source 15 S column (XK16 × 10 cm, GE Healthcare) to remove contaminants and potential endotoxins. After equilibration, mGM-CSF was eluted by a linear gradient over 20 column volumes of NaCl from 0 to 1 M in 25 mM sodium acetate pH 4.5, 0.1% CHAPS. The obtained fractions were analyzed by SDS-PAGE. The major mGM-CSF-containing fractions were pooled and loaded onto a HiLoad 26/60 Superdex 75 prep grade size exclusion column (GE Healthcare) with PBS as the elution buffer. Following concentration of the product peak by ultrafiltration (Vivaspin 20, 3000 MWCO; Sartorius Stedim Biotech), the mGM-CSF concentration was determined using the BCA assay (Pierce) and endotoxin levels were determined with the ToxinSensor Chromogenic LAL Endotoxin Assay Kit (GenScript USA). The process generated 92 mg mGM-CSF from 5 liter of supernatant (18 mg/l) with a purity >95% based on SDS-PAGE and an endotoxin level of 0.15 EU/mg.

### mGM-CSF activity in murine monocyte differentiation into dendritic cells

Bone marrow (BM) cells (C57Bl/6 mice) were cultured for 8 days in dendritic cell (DC) culture medium: RPMI 1640 containing GlutaMAX-I, supplemented with 5% (vol/vol) FCS, 50 μM β-mercaptoethanol and 20 ng/ml mGM-CSF, the latter either produced in *E. coli *(Peprotech) or in-house in *P. pastoris*. Following differentiation, bone marrow-derived DCs (BM-DCs) were infected for 24 h with *Mycobacterium bovis *BCG 1721, a derivative of *M*. *bovis *BCG Pasteur, carrying a non-restrictive rpsL alteration. Following infection, the BM-DCs were collected by centrifuging (10 min at 150*g *and 4°C). After washing in PBS, the cells (both infected and non-infected) were stained for different cell surface markers, *i.e*. CD40-biotin (Pharmingen), CD86-biotin (Pharmingen), followed by streptavidin-PE, CD80-PE (Pharmingen), CD273-PE (Pharmingen), CD274-PE (Pharmingen), CD1d-PE (eBioscience). The expression of these surface markers was checked on the CD11c positive population (a marker for DCs) with CD11c-PE-Cy5 (eBioscience), which was almost 100% in both preparations. Measurements were performed on a FACSCalibur flow cytometer (BD Biosciences) equipped with CellQuest software.

### Analytical techniques

Secreted proteins were TCA precipitated by adding 10% v/v 10 mg/ml deoxycholate and 10% trichloro acetic acid. After centrifugation (20 min at >10.000*g *and 4°C) the protein pellet was dissolved in Laemmli loading buffer supplemented with 1 M unbuffered Tris to neutralize the solution and boiled for 10 minutes. Samples were analyzed by SDS-PAGE using 15% gels casted in-house according to [22]. Proteins were visualized by staining with Coomassie Brilliant Blue (Sigma) or blotted onto nitrocellulose membranes (Hybond-C extra, 45 μm, Amersham Life Science) using the Mini Trans-Blot Cells from Bio-Rad (Hercules, CA, USA). Western blot analysis [23] was performed using a rat anti-mGM-CSF monoclonal antibody (Imgenex) at a concentration of 1 μg/ml (2 hours) and a horseradish peroxidase-conjugated goat anti-rat IgG (Sigma) as the secondary antibody (2 hours). Samples were deglycosylated by treatment with PNGase F (New England Biolabs). N-linked oligosaccharides were analyzed by fluorophore-assisted carbohydrate electrophoresis (FACE), with an ABI 3130 capillary DNA sequencer as described previously [24].

Cell density was expressed as wet cell weight (WCW). This was done by sampling duplicate 10 ml aliquots of the fermentation broth into preweighed 15 ml conical tubes. The samples were centrifuged at 10.000*g*, the supernatants were discarded, and the pellets were weighed. One gram WCW/l is equivalent to ~0.27 g dry cells/l ~ 1 OD_600 _[25].

Murine GM-CSF expression levels were quantified by ELISA (eBioscience). After correction for cell density, yields were expressed as milligram per liter fermentation broth.

## Results and Discussion

### Expression vector construction and GS115mGM-CSF strain generation

**Figure 1:**
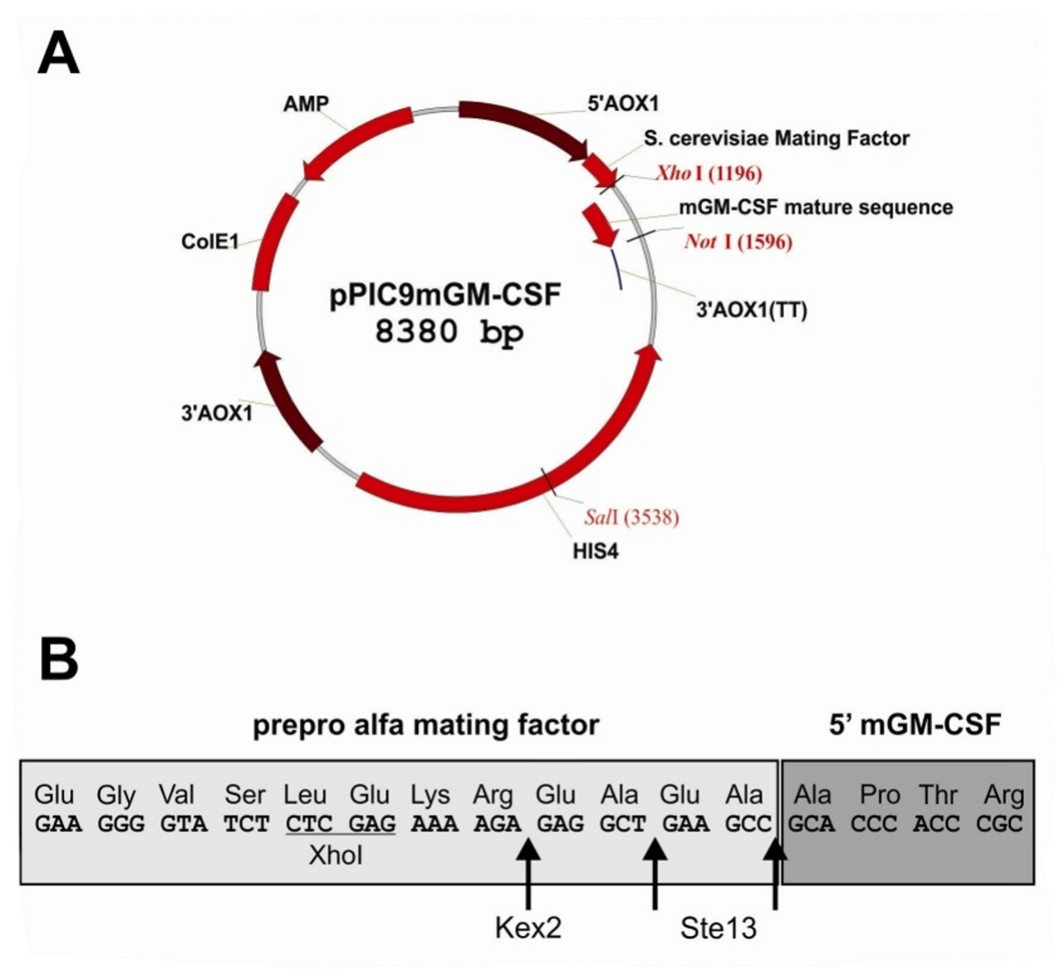
**(A) Schematic representation of the pPIC9mGM-CSF expression vector and (B) Secretion signal processing**. The *S. cerevisiae *α-factor secretion signal is removed by the consecutive action of two enzymes: Kex2p cleaves between the arginine and the glutamic acid residue in the sequence EKREAEA; Ste13p removes both EA repeats.

The sequence encoding the mature mGM-CSF protein was PCR amplified and cloned in the *P. pastoris *pPIC9 expression vector, in-frame with the *S. cerevisiae *α-mating factor prepropeptide coding sequence and under transcriptional control of the *P. pastoris AOX1 *promoter. The resulting vector was termed pPIC9mGM-CSF (Figure 1A). It has been reported that incorporation of Glu-Ala repeats immediately following the Kex2p recognition site can significantly increase Kex2p processing efficiency and final secreted product yield [26]. Therefore, to enhance cleavage of the secretion signal, we inserted two Glu-Ala repeats after the Kex2p recognition site (Figure 1B).

**Figure 2:**
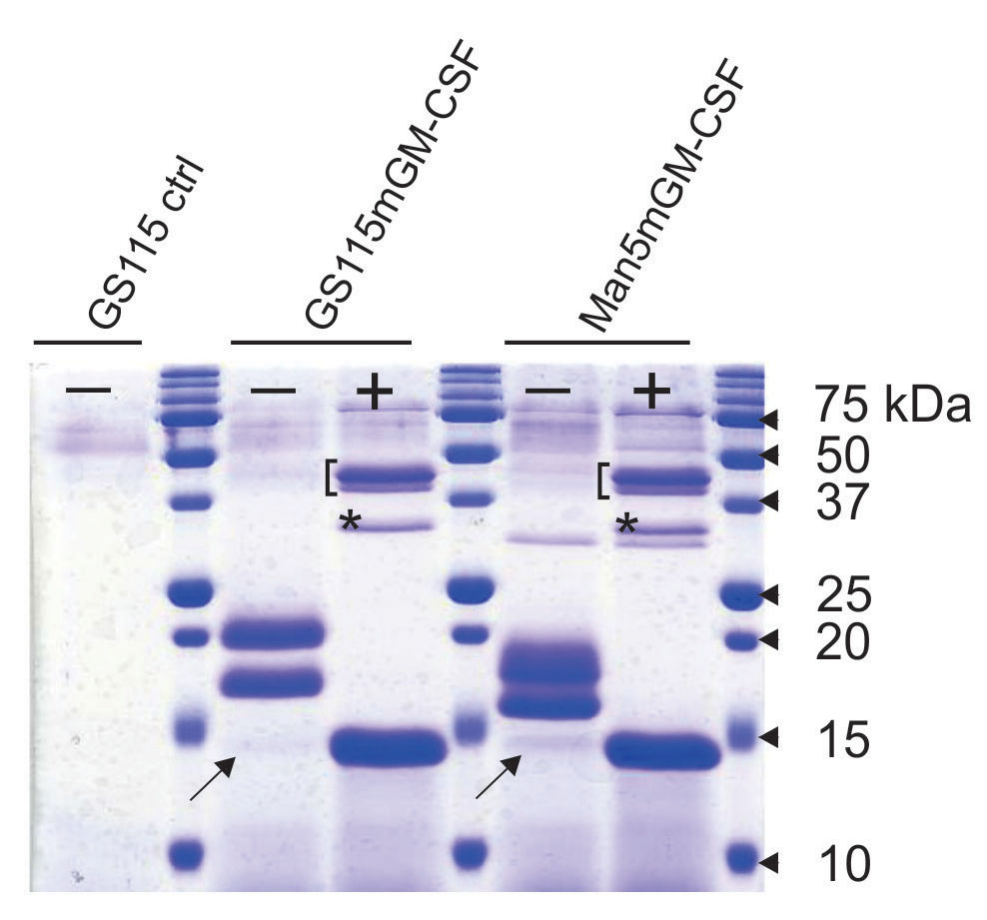
**SDS-PAGE analysis of GS115, GS115mGM-CSF and Man5mGM-CSF culture supernatant untreated (-) or treated with PNGase F (+)**. Mouse GM-CSF appears as three molecular species ranging in molecular mass from approximately 14 to 18 kDa. Treatment with PNGase F showed that the upper two bands correspond to mGM-CSF carrying two and one N-glycans, while the minor lower band (indicated with an arrow) is non-N-glycosylated mGM-CSF. The extra band indicated with an asterisk in both "+" lanes is PNGase F. The two bands marked with a bracket in the "+" lanes are highly N-glycosylated endogenous *P. pastoris *glycoproteins that, due to their extensive and heterogeneous modification with carbohydrates, do not present as one single band on SDS-PAGE. Upon de-N-glycosylation, however, this heterogeneity is eliminated, resulting in two clearly defined bands.

The SalI-linearized pPIC9mGM-CSF vector was subsequently transformed to the wild type *P. pastoris *strain GS115. Twelve individual clones were analyzed by SDS-PAGE for mGM-CSF secretion in the culture supernatant (data not shown). Eleven clones effectively produced mGM-CSF and the one with the highest expression level was withheld for further analysis. This strain was named GS115mGM-CSF. SDS-PAGE analysis showed that mGM-CSF was produced as three molecular species ranging in molecular weight from ~14 to ~18 kDa (Figure 2). After de-N-glycosylation with PNGase F, only one ~14 kDa band remained. Since mGM-CSF has two potential N-glycosylation sites, this indicates that the upper and middle bands correspond to mGM-CSF carrying two and one N-glycans, respectively, while the minor lower band is non-N-glycosylated mGM-CSF (Figure 2). Although we did not perform N-terminal sequencing, the fact that de-N-glycosylated mGM-CSF appeared on SDS-PAGE (Figure 2) and Western blot (data not shown) as a protein of ~14 kDa (which corresponds very well with a theoretical molecular weight of 14.1 kDa) indicates that Kex2p processing was very efficient. After all, non-cleavage of the prepropeptide by Kex2p would have increased the molecular weight by at least 9 kDa (not taking into account the contribution of oligosaccharides attached to the three potential N-glycosylation sites of the prepropeptide). A potential downside of this approach, however, is subsequent incomplete removal of both Glu-Ala repeats by Ste13p, resulting in a final product with two or four additional N-terminal amino acids. We did not determine the extent to which this happened, though, since we were primarily concerned about introducing additional N-glycosylation sites through inefficient Kex2p processing.

### P. pastoris-produced mGM-CSF is biologically active

**Figure 3:**
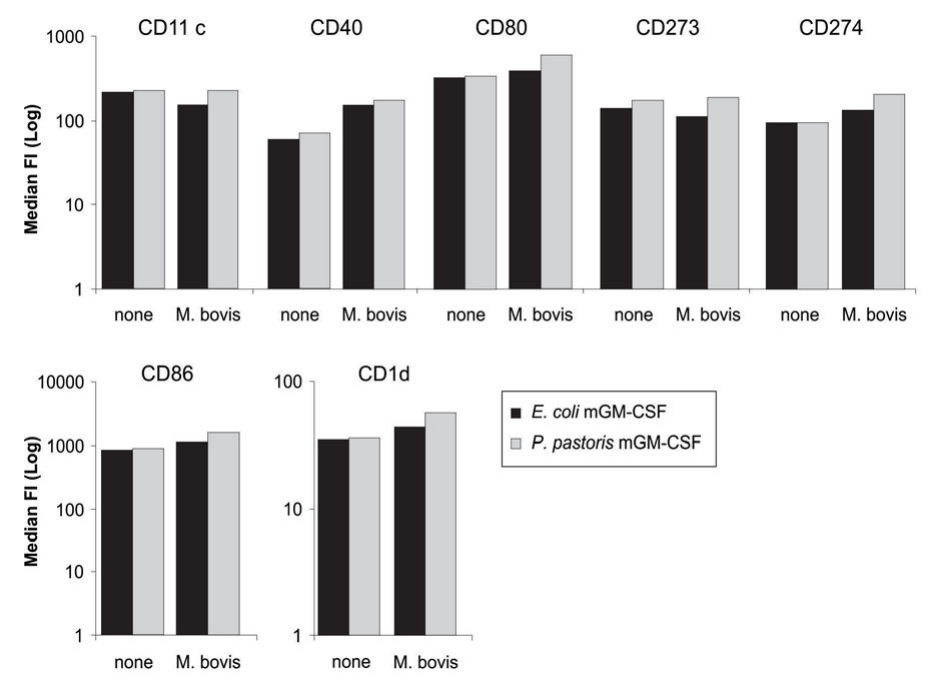
***P. pastoris-*produced mGM-CSF is biologically active**. Mouse bone marrow cells were differentiated into DCs using either *E. coli*- (black bars) or *P. pastoris*-produced (grey bars) mGM-CSF. Six surface maturation markers were measured by flow cytometry on non-infected ('none') or *M. bovis *BCG-infected BM-DCs ('M. bovis'), which were also CD11c+. Both mGM-CSF preparations were equally potent in differentiating bone marrow cells into BM-DCs (similar expression of cell surface markers). The latter also responded similarly to a stimulus, *i.e*., infection with *M. bovis *BCG, regardless of the source of mGM-CSF used for differentiation. The percentage of obtained CD11c+ cells was also very similar for both mGM-CSF sources (almost 100%; data not shown). This demonstrates that, based on cell surface marker expression and reaction to *M. bovis *BCG infection, both sources of mGM-CSF produced phenotypically indistinguishable BM-DCs.

To ascertain that *P. pastoris *is a suitable host organism for the production of biologically active mGM-CSF, the latter was purified from the culture supernatants of shake flask cultures of GS115mGM-CSF to a final yield of ~18 mg/l and an endotoxin content of 0.15 EU/mg. *P*. *pastoris*-produced mGM-CSF was then used head-to-head against commercially available *E. coli*-produced mGM-CSF (Peprotech) to differentiate murine bone marrow-derived monocytes into dendritic cells (BM-DC). Similar expression of 6 differentiation/activation-associated cell surface markers on BM-DCs that were either differentiated with *Pichia*-derived or *E. coli*-derived mGM-CSF demonstrated that both forms are equally potent. Additionally, both sets of BM-DCs responded similarly to an *M. bovis *BCG infection, confirming that differentiation with *P. pastoris*- or *E. coli*-derived mGM-CSF does not induce phenotypic differences during the differentiation process (Figure 3; results shown are representative for three independent experiments). This demonstrates that mGM-CSF produced in *P. pastoris *is as biologically active as the commercial *E. coli *preparation. As a testament to the quality of the *P. pastoris*-derived mGM-CSF, immunologists in our department have switched to utilizing *P. pastoris*-derived mGM-CSF for murine dendritic cell differentiation.

### Man5mGM-CSF strain selection

GlycoSwitch-Man5, a glycoengineered GS115-derived *P. pastoris *strain that predominantly modifies its glycoproteins with Man_5_GlcNAc_2 _N-glycans, was subsequently used as the host strain. Upon transformation of the expression vector pPIC9mGM-CSF to GlycoSwitch-Man5, 18 individual clones were evaluated for mGM-CSF expression by SDS-PAGE of the culture supernatant; 13 (68%) expressed mGM-CSF (data not shown). These clones were designated Man5mGM-CSF. As did the wild type strain, the GlycoSwitch-Man5 strain produced mGM-CSF as three distinct glycoforms, *i.e*., modified with two, one or no N-glycans (Figure 2). As a result of the engineered N-glycosylation pathway, however, both N-glycosylated bands possessed a discernible higher electrophoretic mobility on SDS-PAGE compared to GS115-produced mGM-CSF. De-N-glycosylation with PNGase F completely eliminated this difference in molecular weight, indicating that it was entirely attributable to the presence of different size N-glycans (Figure 2).

**Figure 4:**
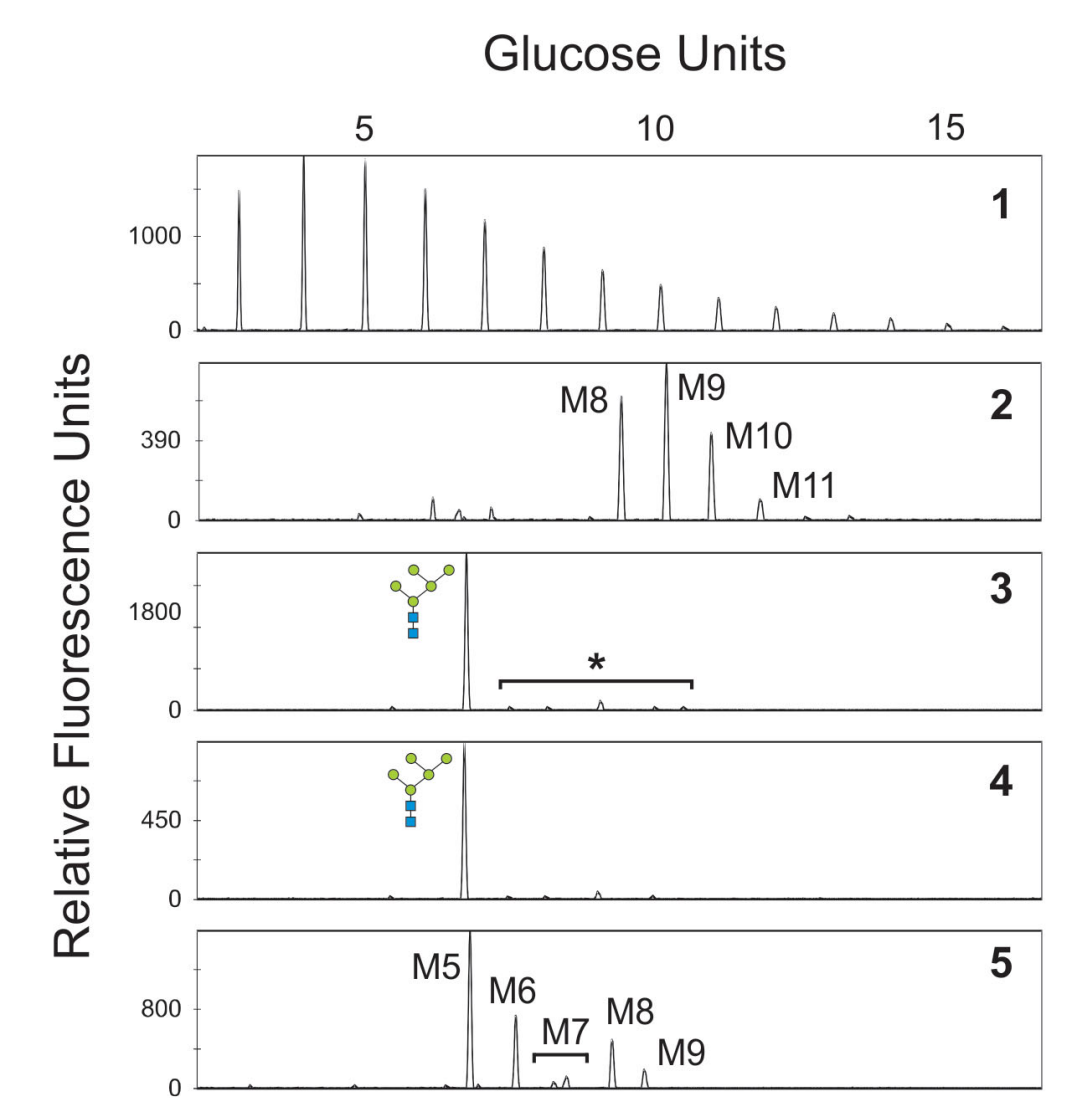
**DSA-FACE analysis of the total N-glycan pool from Man5mGM-CSF strain**. Panel 1 shows the results for a malto-dextrose reference. Panels 2 through 5 show the results for N-glycans, as follows: panel 2: GS115mGM-CSF (the main peak is Man_9_GlcNAc_2 _[M9]); panel 3, Man5mGM-CSF (the predominant peak is Man_5_GlcNAc_2_; a minor fraction of higher molecular weight structures is marked with an asterisk); panel 4, same as panel 3 but the N-glycans were treated with α-1,2-mannosidase; panel 5, reference N-glycans from bovine RNase B (Man_5-9_GlcNAc_2 _[M5-M9]).

Next, for each Man5mGM-CSF clone, the N-glycans on secreted glycoproteins were analyzed by DSA-FACE. Based on mGM-CSF expression levels and N-glycan homogeneity, one clone was withheld for further analysis. Figure 4 shows DSA-FACE profiles for the selected Man5mGM-CSF clone. The predominant (>90%) N-linked glycan was Man_5_GlcNAc_2 _(Figure 4, panel 3). For comparison, glycoproteins secreted by wild type *P*. *pastoris *(GS115mGM-CSF) were mostly modified with a heterogeneous array of high-mannose-type N-glycans (Figure 4, panel 2). A minor fraction (<10%) of the total N-glycan pool secreted by Man5mGM-CSF was composed of higher molecular weight structures (marked with an asterisk in Figure 4, panel 3). For the majority of these peaks, however, *in vitro *α-1,2-mannosidase digestion did not cause a shift in mobility, indicating that these peaks were not the result of incomplete trimming by the introduced *T. reesei *α-1,2-mannosidase (in Figure 4, compare panels 3 and 4).

### Maximum specific growth rate (μ_max_) under methanol excess conditions

To determine μ_max _of Man5mGM-CSF when methanol is in excess, bioreactor fermentations were performed with a constant methanol concentration (S) of ~2 g/l. Only minor fluctuations in methanol concentration were observed throughout the fermentations. μ_max _and ν_MeOH,max _were 0.0624 h^-1 ^and 0.0743 g/g WCW/h, respectively. This corresponds to a Y_X/S _(yield of biomass to substrate) of 0.8398 g/g. For comparison, the 'empty' GlycoSwitch-Man5 strain, *i.e*., GlycoSwitch-Man5 transformed with pPIC9, had a μ_max _and ν_MeOH,max _of 0.0816 h^-1 ^and 0.052 g/g WCW/h, respectively, corresponding to a Y_X/S _value of 1.569 g/g. These data illustrate the burden that mGM-CSF expression places on the cells: μ_max _of Man5mGM-CSF is about 76% of μ_max _of Man5. Moreover, under excess methanol conditions, Man5mGM-CSF is about 50% less efficient in converting methanol to biomass than GlycoSwitch-Man5.

### Methanol-limited cultures

By substitution of μ_max _and ν_MeOH,max _into equations (1) and (2), the methanol feed rate, F, needed to achieve a desired μ could be estimated by the following equation:

(3) $$\text{F}=1.1907\mu ({\text{X}}_{0}{\text{V}}_{0}){\text{e}}^{\mu \text{t}}.$$

**Figure 5:**
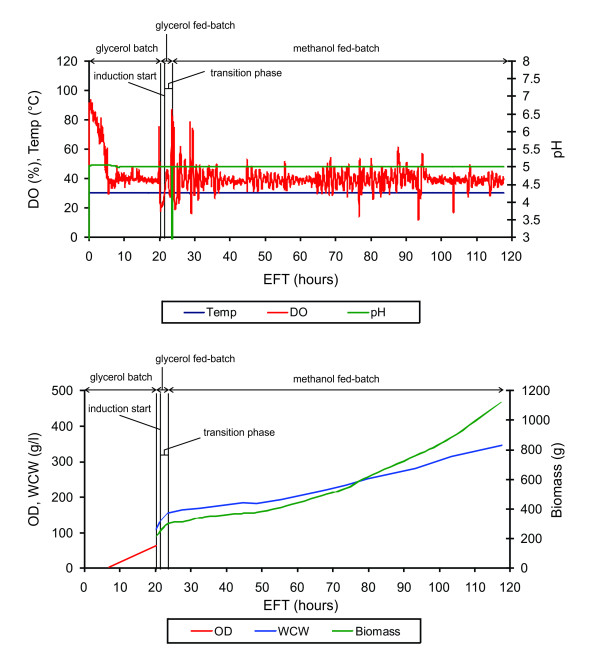
**Fermentation of Man5mGM-CSF at 0.015 h^-1 ^(~25% of μ_max_)**. Plotted parameters show dissolved oxygen (DO), pH, temperature, OD_600_, WCW and biomass during 70 hours of fermentation. The initial batch was grown in FM22 media containing 40 g/l glycerol. Following an increase in DO due to carbon source limitation at around 20 h EFT, the glycerol fed-batch process was initiated. Glycerol was added at 20 g/l/h for 1 hour. The transition phase was initiated by the addition of 1.5 g/l methanol. Simultaneously, the glycerol feed rate was ramped down linearly from 20 g/l/h to 0 over a 3-h period. By the end of the transition phase, the cells were fully adapted to methanol, which was confirmed by a sharp drop in DO. At this point, methanol feeding was initiated. DO was maintained at 40%.

The desired values for μ were set to 0.0156 h^-1 ^(25% μ_max_), 0.0312 h^-1 ^(50% μ_max_) and 0.0468 h^-1 ^(75% μ_max_). X_0 _and V_0 _were determined just prior to the start of the methanol feed profile. The actual μ values were 0.015 h^-1^, 0.0332 h^-1^, and 0.0459 h^-1^, which correlated very well to the desired values. Figure 5 shows the evolution of several key parameters over time for the fermentation of Man5mGM-CSF at 0.015 h^-1 ^(~25% of μ_max_).

### mGM-CSF yield

**Figure 6:**
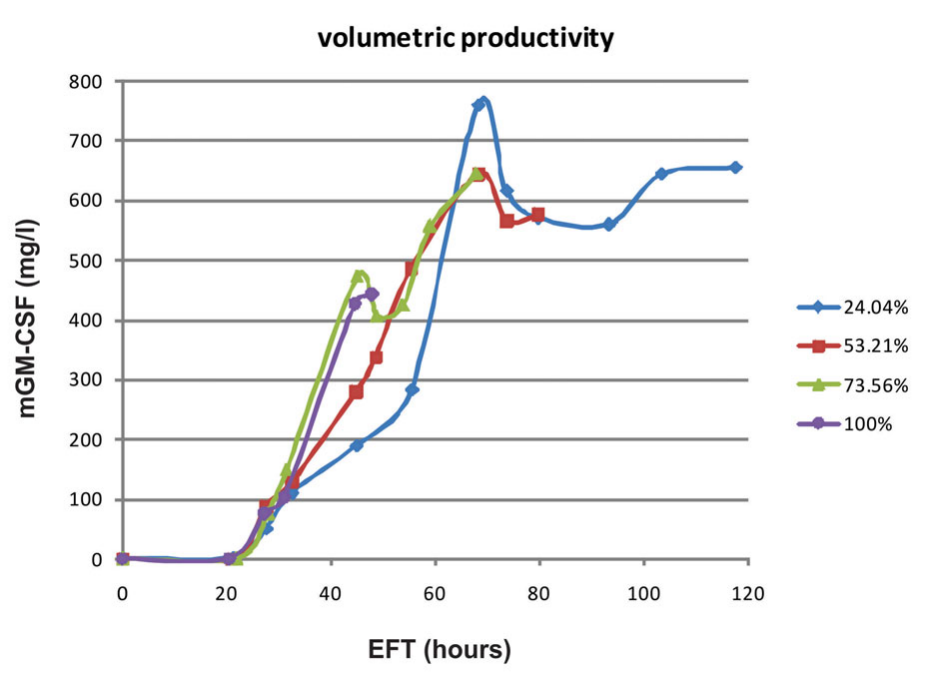
**Typical time course of mGM-CSF production under different specific growth rates**. Growth rates are shown as a percentage of μ_max_. Mouse GM-CSF yields are expressed as milligram per liter fermentation broth and were corrected for cell density.

Protein production kinetics of the four fed-batch fermentation types are shown in Figure 6. Murine GM-CSF yield per unit volume of fermentation broth (*i.e*., after correction for cell density) was maximum by growing the cells at μ = 0.015 h^-1 ^(24.04% μ_max_) during the induction phase. In this way, a volumetric yield of 760 mg/l could be achieved after ~68 h of fermentation. Although volumetric yields decreased slightly after this time point, total mGM-CSF levels continued to increase during the entire course of the fermentation. Higher growth rates resulted in decreased yields (Table 1). Both growth at μ = 0.0332 h^-1 ^(53.21% μ_max_) and μ = 0.0459 h^-1 ^(73.56% μ_max_) decreased yields by about 15.3%. Allowing the cells to achieve their maximum growth rate under methanol conditions (μ_max _= 0.0624 h^-1^) further decreased mGM-CSF yields by as much as 42% (442 mg/l). Hence, limiting the growth rate during the induction phase to ~25% of μ_max _resulted in maximum mGM-CSF yield. During the entire course of fermentation, total recombinant mGM-CSF levels kept increasing regardless of the specific growth rate (data not shown), indicating that proteolytic degradation was not a significant problem. This is in contrast to many other recombinant proteins produced in *P*. *pastoris *that get increasingly degraded during the fermentation [27,28].

**Table 1:** Effect of various specific growth rates on mGM-CSF production.

Specific growth **rate, μ (h**^**-1**^**)**	Elapsed fermentation time (h)	Wet cell density (g/l)	Max mGM-CSF **yield**^**a **^**(mg/l)**
0.0624	47.76	368.3	442.7
0.0459	67.99	448	644.0
0.0332	68.25	370.25	644.4
0.015	68.25	221.4	760.4

^a ^Refers to mGM-CSF yield per unit volume of the fermentation broth.

### N-glycan analysis

**Figure 7:**
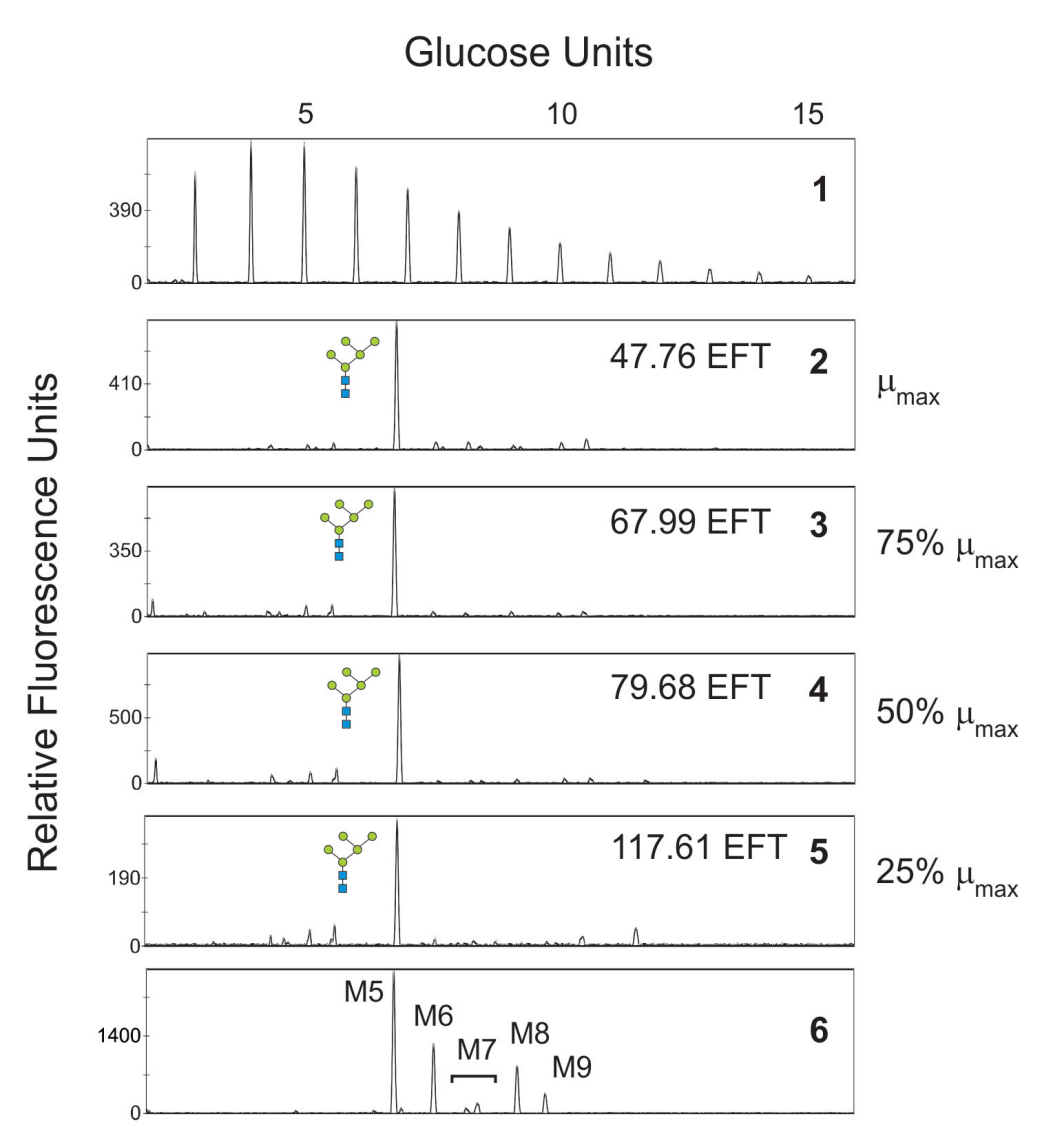
**DSA-FACE N-glycan analysis at the end of the fermentation**. Panel 1 shows the results for a malto-dextrose reference. Panels 2 through 6 show the results for N-glycans, as follows: panel 2, growth at μ_max_; panel 3, growth at 75% μ_max_; panel 4, growth at 50% μ_max_; panel 5, growth at 25% μ_max_; panel 6, reference N-glycans from bovine RNase B (Man_5-9_GlcNAc_2 _[M5-M9]). For each sample, the EFT sampling time point is indicated.

Fermentation supernatant samples were analyzed by DSA-FACE (Figure 7). The predominant peak during the entire course of all fermentations was Man_5_GlcNAc_2_. Even after >100 hours of fermentation, the level of N-glycan homogeneity was comparable to the level obtained in small scale cultures [2]. Since all fermentations were performed under non-selective conditions, this illustrates the stability of the Man5mGM-CSF strain after multiple generations.

Regardless of the specific growth rate, DSA-FACE analysis revealed the presence of low-abundance peaks. Several of these derive from the cultivation medium (not shown), but there is a small fraction (<10%) of larger N-glycan species corresponding to Man_6-10_GlcNAc_2 _structures. These oligosaccharides are believed to be the result of 1) incomplete processing by the introduced α-1,2-mannosidase-HDEL, 2) interfering endogenous mannosyltransferases, or 3) a combination of those two. Importantly though, growth under fermentation conditions did not decrease the conversion efficiency of *P. pastoris *hypermannosyl-type N-glycans to Man_5_GlcNAc_2 _structures as compared to small scale cultivation experiments, again illustrating the robustness of this strain.

## Conclusions

In this paper, we describe a basic but robust fed-batch fermentation protocol for the secreted production of murine GM-CSF in a glycoengineered *P. pastoris *strain that predominantly modifies its glycoproteins with Man_5_GlcNAc_2 _N-glycans. We have shown that growth rate does not significantly affect N-glycan homogeneity: regardless of the specific growth rate the predominant peak during the entire course of all fermentations was Man_5_GlcNAc_2_. There was, however, a clear relationship between product yield and specific growth rate. Cultivation of the cells at ~25% of their maximal growth rate (μ = 0.015 h^-1^) resulted in a volumetric yield of ~760 mg mGM-CSF per liter fermentation broth after ~68 h of fermentation. Higher specific growth rates during the induction phase resulted in decreased mGM-CSF yields.

To date, only one similar study has been published. In this paper, researchers at GlycoFi Inc. (a wholly-owned subsidiary of Merck & Co Inc.) describe a strain capable of producing >1g/l of human IgG1 with >90% homogeneous Man_5_GlcNAc_2 _N-glycans across a range of fermentation conditions [29]. Unfortunately, the report did not include N-glycan analysis data, which precludes a more direct comparison with our data.

However, it is clear from both reports that conversion of the *P. pastoris *N-glycosylation pathway to the Man_5_GlcNAc_2 _structure does not affect the yeast's physiology much and that such engineered strains are fully capable of producing diverse mammalian glycoproteins at high levels, with the desired homogenous N-glycosylation. As the Man_5_GlcNAc_2 _structure is the starting point for buildup of mammalian complex-type N-glycans, future studies will address the behaviour of more extensively engineered strains in high cell density cultivation.

## List of Abbreviations

EFT: elapsed fermentation time (h); F: methanol feed rate (g/h); S: methanol concentration (g/L); T: Culture time (h); V: broth volume (L); WCW: wet cell weight; X: cell density (g WCW/L); Y_X/S_: Yield of biomass to substrate (g/g); μ: Specific growth rate under methanol conditions; μ_max_: maximum specific growth rate under methanol conditions; ν_max_: maximum specific methanol consumption rate (g/g/h)

## Declaration of competing interests

The authors MI, NF, JH, AVH and MMM declare that they have no competing interests. PPJ, RC and NC are either inventors or share otherwise in proceeds of licensing of patents and patent applications covering parts of the described GlycoSwitch technology.

## Authors' contributions

PPJ constructed the mGM-CSF expression vector, generated and analyzed the *P. pastoris *strains, performed the fermentations, interpreted the glycan analysis data and drafted the manuscript. MI assisted in designing the fermentation strategy. NF performed the monocyte differentiation assay and edited the manuscript. JH purified the *Pichia*-produced mGM-CSF and edited the manuscript. AVH was responsible for N-glycan analysis. MMM supervised the fermentations and edited the manuscript. RC conceived and initiated the study. NC supervised experiments and reviewed the final manuscript. All authors read and approved the final manuscript.
